# Bioactive Lipid Mediator Resolvin D5 Suppresses Melanogenesis in α-MSH-Stimulated B16F10 Cells via ROS-Associated p38/β-Catenin/MITF Regulation

**DOI:** 10.4014/jmb.2604.04013

**Published:** 2026-06-22

**Authors:** Hyeok Jin Choi, So Jung Park, Jeong Won Choi, Gyeong Eun Im, Hwan Lee, Seongmin Choi, Deok-Kun Oh, Kyung-Chul Shin, Jin Boo Jeong

**Affiliations:** 1 Department of Forest Science, Gyeongkuk National University, Andong 36729, Republic of Korea; 2 Sphere Corporation Food Research Center, Seoul 06159, Republic of Korea; 3 Department of Bioscience and Biotechnology, Konkuk University, Seoul 05029, Republic of Korea; 4 Department of Bioscience and Biotechnology, Hankuk University of Foreign Studies, Yongin-si 17035, Republic of Korea

**Keywords:** Resolvin D5, Melanogenesis, B16F10 cells, ROS, p38 MAPK, MITF

## Abstract

Excessive melanin production contributes to hyperpigmentation disorders, highlighting the need for safer, mechanism-based depigmenting agents. This study investigated the anti-melanogenic effects of Resolvin D5 (RVD5) in α-MSH-stimulated B16F10 melanoma cells. RVD5 significantly reduced extracellular and intracellular melanin levels without compromising cell viability or cell number. Mechanistically, RVD5 suppressed tyrosinase expression and activity, accompanied by downregulation of MITF and β-catenin. RVD5 also dose-dependently inhibited α-MSH-induced phosphorylation of ERK1/2, p38, and JNK, while pharmacological inhibitor analysis suggested that p38 is a functionally dominant MAPK branch contributing to melanin output. In parallel, RVD5 reduced intracellular ROS levels, and the ROS scavenger NAC mimicked several effects of RVD5, supporting the involvement of ROS-associated signaling. Overall, RVD5 attenuates melanogenesis through antioxidant action, broad MAPK attenuation, and downregulation of the β-catenin/MITF/tyrosinase regulatory pathway.

## Introduction

Skin pigmentation is an essential photoprotective process in which melanin is synthesized and distributed to the epidermis to defend against ultraviolet (UV) radiation [1,2]. However, excessive melanin production contributes to various hyperpigmentation disorders such as melasma, solar lentigines, and post-inflammatory hyperpigmentation, which can negatively affect both aesthetic appearance and quality of life [3]. Despite the availability of depigmenting agents like hydroquinone, kojic acid, and arbutin, their clinical use is limited due to cytotoxicity, instability, or potential side effects on skin integrity [4]. This underscores the need to develop safe, stable, and mechanistically validated compounds that regulate melanogenesis effectively [5].

Melanin biosynthesis is primarily regulated by the microphthalmia-associated transcription factor (MITF), which controls the expression of the key melanogenic enzymes tyrosinase, tyrosinase-related protein-1 (TRP-1), and TRP2 [6]. MITF activity is tightly regulated by upstream signaling pathways. The MAPK family, particularly p38, is activated in response to α-melanocyte-stimulating hormone (α-MSH) and oxidative stress and has been shown to promote MITF transcription and function [7, 8]. In parallel, the Wnt/β-catenin pathway enhances melanogenesis by directly activating the MITF promoter via β-catenin/LEF1 complexes [9]. Thus, both p38 and β-catenin signaling contribute to the transcriptional control of melanogenesis through MITF. Recently, reactive oxygen species (ROS) have been recognized as critical modulators of melanogenic signaling [10]. In melanocytes, ROS are generated under UV exposure or hormonal stimulation and contribute to the activation of MAPKs including p38, which results in promoting melanin production [11, 12]. Consequently, targeting ROS-mediated signaling presents a compelling strategy for the upstream melanogenic regulation.

Resolvin D5 (RVD5) is a specialized pro-resolving mediator derived from docosahexaenoic acid and is known to play key roles in the resolution of inflammation and attenuation of oxidative stress [13]. In dermatological contexts, RVD5 has been reported to protect UVB-exposed skin by modulating redox and inflammatory signaling [14], but its role in melanogenesis has not been elucidated. Whether RVD5 can regulate melanogenic signaling pathways such as ROS/p38/β-catenin/MITF/tyrosinase in pigment-producing cells remains an open question.

In this study, we investigated the anti-melanogenic effects of RVD5 in α-MSH-stimulated B16F10 melanoma cells, a well-established model for melanogenesis. We demonstrate that RVD5 exerts dose-dependent antioxidant effects, reduces intracellular ROS accumulation, broadly attenuates α-MSH-induced MAPK phosphorylation, and downregulates β-catenin, MITF, and tyrosinase expression. These molecular changes culminate in the downregulation of tyrosinase expression and a marked decrease in both intracellular and extracellular melanin production. Furthermore, our pharmacological data suggest that RVD5 suppresses melanogenesis through ROS-associated MAPK modulation and downregulation of the β-catenin/MITF/tyrosinase regulatory pathway, offering a candidate for mechanistically guided depigmentation strategies.

## Materials and Methods

### Chemical Reagents

Resolvin D5 (RVD5; Cat. no. 10007280) was obtained from Cayman Chemical (USA). Alpha-melanocyte stimulating hormone (α-MSH; Cat. no. M4135), 3-(4,5-dimethylthiazol-2-yl)-2,5-diphenyltetrazolium bromide (MTT; Cat. no. 475989), PD98059 (ERK1/2 inhibitor; Cat. no. 513000), SB203580 (p38 inhibitor; Cat. no. 58307), SP600125 (JNK inhibitor; Cat. no. 55567), N-acetyl-L-cysteine (NAC; ROS inhibitor; Cat. no. A9165), and 2,2-diphenyl-1-picrylhydrazyl (DPPH; Cat. no. D9132) were purchased from Sigma-Aldrich (USA). Primary antibodies against ERK1/2 (Cat. no. 9102), phospho-ERK1/2 (Cat. no. 4377), p38 (Cat. no. 9212), phospho-p38 (Cat. no. 4511), JNK (Cat. no. 9258), phospho-JNK (Cat. no. 9251), β-catenin (Cat. no. 9562), and β-actin (Cat. no. 5125) were supplied by Cell Signaling Technology (USA). Antibodies for tyrosinase Cat. no. sc-20035) and microphthalmia-associated transcription factor (MITF; Cat. no. MAB3747) were obtained from Santa Cruz Biotechnology (USA) and Merck Millipore (USA), respectively. HRP-conjugated anti-rabbit IgG (Cat. no. 7074) and anti-mouse IgG (Cat. no. 7076) secondary antibodies were also purchased from Cell Signaling Technology.

### Cell Culture

B16F10 murine melanoma cells (Cat. no. CRL-6475) were purchased from the American Type Culture Collection (ATCC, USA). Cells were grown in DMEM/F-12 containing 10% heat-inactivated fetal bovine serum, 100 U/mL penicillin, and 100 μg/mL streptomycin, and maintained at 37°C in a humidified 5% CO_2_ atmosphere. Fresh medium was supplied every 2-3 days. When cultures reached approximately 70-80% confluence, cells were detached with 0.05% trypsin-EDTA and passaged. To minimize phenotypic drift, only cells within 15 passages were used for the experiments.

### Cell Viability Assay

Cell viability following RVD5 exposure was assessed using the MTT colorimetric assay. B16F10 cells were seeded into 96-well plates at 1 × 10^4^ cells/well and allowed to attach for 24 h. Cells were then incubated with the indicated concentrations of RVD5, whereas control cells received an equivalent amount of DMSO. After 48 h, MTT was added to each well to a final concentration of 0.5 mg/mL, and the plates were incubated for an additional 4 h at 37°C. The medium was removed, the formazan crystals were dissolved in DMSO, and absorbance was recorded at 570 nm using a SpectraMax M2 microplate reader (Molecular Devices, USA).

### Cell Number Measurement

B16F10 cells were seeded in 6-well plates and allowed to attach overnight at 37°C in a humidified atmosphere containing 5% CO_2_. The cells were then treated with RVD5 at 25 or 50 μg/mL for 48 h. After treatment, the cells were harvested, and total cell number was measured using a NucleoCounter NC-250 instrument (Chemometec, Denmark) according to the manufacturer’s protocol.

### Measurement of Extracellular and Intracellular Melanin Contents

Melanin production was determined by measuring both secreted and cellular melanin in α-MSH-stimulated B16F10 cells. Cells were plated in 6-well plates at 2 × 10^5^ cells/well and cultured overnight. The next day, cells were pre-incubated for 2 h with RVD5, PD98059, SB203580, SP600125, or NAC, followed by co-treatment with α-MSH (1 μg/mL) for 48 h. For extracellular melanin analysis, conditioned medium was collected and centrifuged at 12,000 × g for 10 min to remove particulate material. The absorbance of the clarified supernatant was measured at 405 nm with a SpectraMax M2 instrument. For intracellular melanin determination, cells were washed twice with PBS, detached with trypsin-EDTA, and pelleted by centrifugation at 12,000 × g for 5 min. The pellets were dissolved in 1 N NaOH containing 10% DMSO at 80°C for 1 h, and absorbance was then measured at 405 nm.

### Measurement of the Cellular Tyrosinase Activity

Intracellular tyrosinase activity was analyzed in B16F10 cells treated with RVD5 under α-MSH stimulation. Cells were seeded in 6-well plates at 2 × 10^5^ cells/well, incubated overnight, pretreated with RVD5 for 2 h, and then exposed to α-MSH (1 μg/mL) for 48 h. After treatment, cells were washed twice with PBS, harvested with trypsin-EDTA, and centrifuged at 12,000 × g for 10 min. The resulting pellet was lysed in 0.5% Triton X-100 for 30 min at 4°C and centrifuged again at 12,000 × g for 10 min. Protein concentrations in the supernatants were quantified using the bicinchoninic acid (BCA) assay (Pierce, USA). For the enzyme reaction, 80 μL of lysate containing 30 μg protein was combined with 20 μL of 10 mM L-DOPA and incubated at 37°C for 1 h. Dopachrome formation was monitored at 405 nm with a SpectraMax M2 reader, and tyrosinase activity was normalized to total protein.

### DPPH Radical Scavenging Assay

The free radical-scavenging capacity of RVD5 was examined using the DPPH assay. A 0.2 mM DPPH solution was prepared in methanol, and RVD5 was diluted to the required concentrations in the same solvent. Aliquots of RVD5 solution (40 μL) were mixed with 160 μL of DPPH solution and allowed to react for 30 min at room temperature. Absorbance was then measured at 405 nm using a SpectraMax M2 reader.

### Measurement of Cellular ROS Contents

Intracellular ROS levels were measured in α-MSH-stimulated B16F10 cells after RVD5 treatment. Cells were seeded in 6-well plates at 2 × 10^5^ cells/well and allowed to attach overnight. After 2 h of pretreatment with RVD5, cells were incubated with α-MSH (1 μg/mL) for 48 h. ROS generation was then determined using the Intracellular ROS Assay Kit (CELL BIOLABS, INC., USA) according to the manufacturer's instructions.

### Reverse Transcription Polymerase Chain Reaction (RT-PCR)

For RT-PCR analysis, B16F10 cells were seeded in 6-well plates at 2 × 10^5^ cells/well, incubated overnight, pretreated with RVD5 for 2 h, and subsequently stimulated with α-MSH (1 μg/mL) for 48 h. Total RNA was isolated using the RNeasy Mini Kit (Qiagen, Germany). RNA quantity and purity were evaluated from the A260/A280 ratio using a GeneQuant 1300 spectrophotometer (Biochrom, USA). Complementary DNA was synthesized from 1 μg total RNA using the Verso cDNA Kit (Thermo Fisher Scientific, USA). PCR amplification was carried out with primers specific for tyrosinase and GAPDH, which served as the internal reference. Primer sequences were as follows: tyrosinase forward, 5′-GACGGTCACTGCAGACTTTG-3′; tyrosinase reverse, 5′-GCCATGACCAGGATGAC-3′; GAPDH forward, 5′-TGAAGGTCGGTGTGAA CGGATTTCGC-3′; and GAPDH reverse, 5′-CATGTAGGCCATGAGGTCCACCAC-3′. PCR products were separated by agarose gel electrophoresis, visualized using a gel documentation system, and quantified with UN-SCAN-IT gel software version 5.1 (Silk Scientific Inc., USA).

### SDS-PAGE and Western Blot Analysis

B16F10 cells were plated in 6-well plates at 2 × 10^5^ cells/well and cultured overnight. Cells were then pretreated for 2 h with RVD5, SB203580, or NAC, followed by co-exposure to α-MSH (1 μg/mL) for 48 h. After treatment, cells were washed with ice-cold PBS and lysed in RIPA buffer (Thermo Fisher Scientific) supplemented with protease and phosphatase inhibitors (Sigma-Aldrich). Lysates were kept on ice for 30 min and centrifuged at 12,000 × g for 30 min at 4°C. Protein concentrations in the supernatants were determined using the Pierce BCA Protein Assay Kit. Equal amounts of protein (30 μg) were separated on 10-12% SDS-polyacrylamide gels and transferred to nitrocellulose membranes. Membranes were blocked for 1 h at room temperature with 5% non-fat dry milk in TBST and then incubated overnight at 4°C with primary antibodies against tyrosinase, MITF, β-catenin, phospho-p38, p38, phospho-ERK1/2, ERK1/2, phospho-JNK, JNK, and β-actin. After washing with TBST, membranes were incubated with HRP-conjugated secondary antibodies for 1 h at room temperature. Immunoreactive bands were detected using ECL Select Western Blotting Detection Reagent (cat. no. RPN2232; Cytiva, USA) and visualized with an LI-COR C-DiGit Blot Scanner (LI-COR Biosciences, USA). Band intensities were quantified using UN-SCAN-IT gel analysis software version 5.1 and normalized to β-actin.

### Statistical Analysis

All experiments were performed independently at least three times. Data are expressed as mean ± standard deviation (SD). Statistical analyses were conducted using GraphPad Prism version 5.0 (GraphPad Software, Inc.). Differences among groups were evaluated by one-way analysis of variance (ANOVA) followed by Bonferroni's post hoc test, and *p* < 0.05 was considered statistically significant.

## Results

### RVD5 Suppresses α-MSH-Induced Melanogenesis in B16F10 Cells without Affecting Viability

**Fig. 1:**
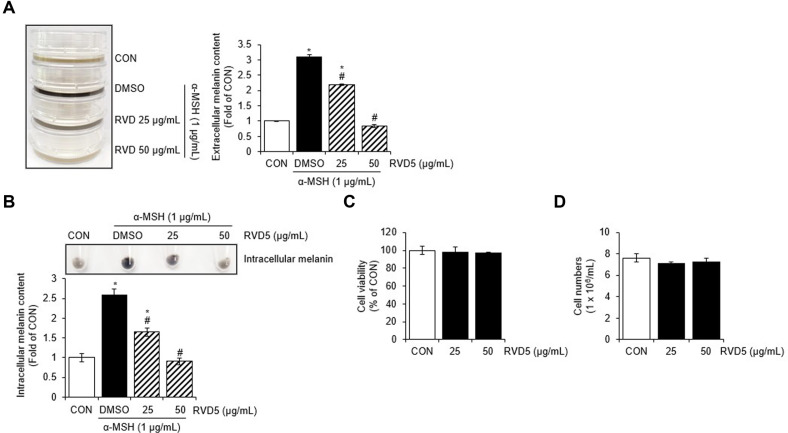
Effects of RVD5 on α-MSH-stimulated melanogenesis and cell viability in B16F10 cells. B16F10 cells were pretreated with RVD5 for 2 h and then co-treated with α-MSH (1 μg/mL) for 48 h. (**A**) Extracellular melanin content was analyzed by measuring the absorbance of the cell culture medium at 405 nm. (**B**) Intracellular melanin content was analyzed by measuring the absorbance of the cell lysates at 405 nm. (**C**) B16F10 cells were treated with RVD5 for 48 h. Cell viability was measured by the MTT assay. (**D**) B16F10 cells were treated with RVD5 at 25 or 50 μg/mL for 48 h, and total cell number was measured using a NucleoCounter NC-250 instrument. * *P* < 0.05 vs CON (untreated group). ^#^ *P* < 0.05 vs DMSO (α-MSH-only treated group).

To determine whether RVD5 modulates melanogenesis, B16F10 cells were stimulated with α-MSH and treated with RVD5. Quantification of extracellular melanin revealed a clear, concentration-dependent reduction in α-MSH-induced melanin release upon RVD5 treatment (Fig. 1A). Consistently, measurement of intracellular melanin showed that RVD5 also decreased the α-MSH-driven accumulation of cellular melanin in a dose-dependent manner across the tested concentration range (Fig. 1B). Importantly, RVD5 did not alter B16F10 cell viability under the same experimental conditions, as assessed by the MTT assay (Fig. 1C). To further verify that the anti-melanogenic effect of RVD5 was not associated with reduced cell number, we additionally measured cell number using an automated cell counting system. Consistent with the MTT results, RVD5 treatment at 25 and 50 μg/mL did not significantly alter cell number compared with the untreated control (Fig. 1D). These findings further support that RVD5 suppresses melanogenesis without reducing cell number. Thus, the anti-melanogenic effects of RVD5 on both extracellular and intracellular melanin are not attributable to cytotoxicity but instead reflect a specific suppression of α-MSH-evoked melanogenic responses. Collectively, these findings identify RVD5 as a non-cytotoxic inhibitor of α-MSH-induced melanogenesis in B16F10 cells.

### RVD5 Downregulates the β-Catenin/MITF/tyrosinase Axis and Suppresses Tyrosinase Activity in α-MSH–Stimulated B16F10 Cells

**Fig. 2:**
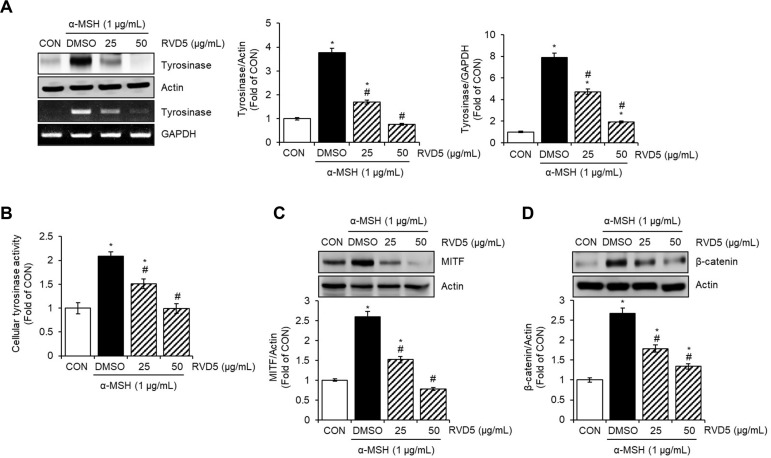
Effects of RVD5 on tyrosinase expression/activity and upstream regulators (MITF and β-catenin) in α-MSH-stimulated B16F10 cells. B16F10 cells were pretreated with RVD5 for 2 h and then co-treated with α-MSH (1 μg/mL) for 48 h. (**A**) The levels of tyrosinase protein and mRNA were analyzed using Western blot analysis and RT-PCR, respectively. (**B**) 30 μg of cellular protein was mixed with 10 mM L-DOPA solution at 37°C for 1 h. The absorbance was measured at 405 nm. (**C**) The level of MITF protein was analyzed using Western blot analysis. (**D**) The level of β-catenin protein was analyzed using Western blot analysis. * *P* < 0.05 vs CON (untreated group). ^#^ *P* < 0.05 vs DMSO (α-MSH-only treated group).

To determine whether RVD5 attenuates the melanogenic machinery downstream of α-MSH, we first quantified tyrosinase expression. RVD5 reduced both tyrosinase protein and mRNA levels in α-MSH-stimulated B16F10 cells (Fig. 2A). We next asked whether decreased expression translated into functional inhibition. Consistent with the expression data, RVD5 suppressed cellular tyrosinase activity in α-MSH-challenged cells (Fig. 2B). Because tyrosinase expression is governed by MITF, we examined upstream transcriptional control. RVD5 decreased MITF protein under α-MSH stimulation (Fig. 2C). Given that Wnt/β-catenin signaling positively regulates MITF transcription, we assessed β-catenin. RVD5 lowered β-catenin protein levels in α-MSH-treated cells (Fig. 2D).

### RVD5 Attenuates α-MSH-Induced MAPK Activation, with p38 Showing a Dominant Functional Contribution to Extracellular Melanin Production

**Fig. 3:**
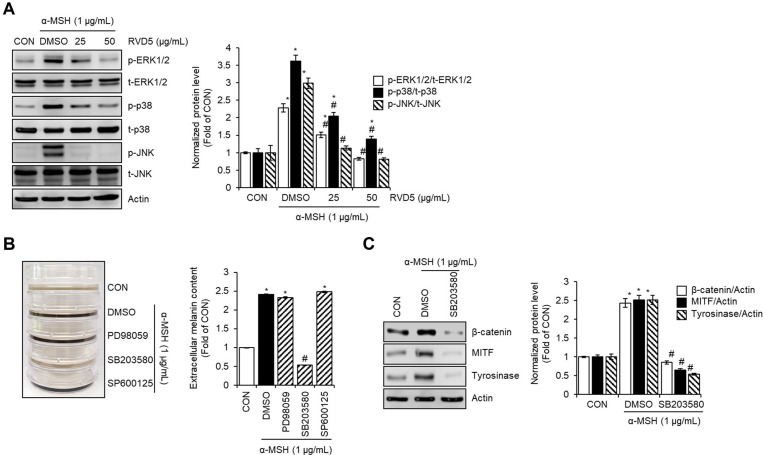
Involvement of MAPK signaling in the anti-melanogenic effect of RVD5 in α-MSH-stimulated B16F10 cells. (**A**) B16F10 cells were pretreated with RVD5 for 2 h and then co-treated with α-MSH (1 μg/mL) for 48 h. The phosphorylation levels of ERK1/2, p38, and JNK were analyzed by Western blotting. (**B**) To identify which MAPK pathway is functionally involved in melanin production, B16F10 cells were pretreated with PD98059 (ERK1/2 inhibitor, 10 μM), SB203580 (p38 inhibitor, 10 μM), or SP600125 (JNK inhibitor, 10 μM) for 2 h and then co-treated with α-MSH (1 μg/mL) for 48 h. Extracellular melanin content was measured at 405 nm. (**C**) To further examine melanogenesis-related proteins associated with p38 inhibition, B16F10 cells were pretreated with SB203580 (10 μM) for 2 h and then co-treated with α-MSH (1 μg/mL) for 48 h. The protein levels of β-catenin, MITF, and tyrosinase were analyzed by Western blotting. * *P* < 0.05 vs. CON (untreated group). ^#^ *P* < 0.05 vs. DMSO (α-MSH-only treated group).

To explore the upstream mechanisms by which RVD5 modulates melanogenesis, we first examined the activation status of MAPKs in α-MSH-stimulated B16F10 cells. RVD5 treatment resulted in a dose-dependent reduction in the phosphorylation of ERK1/2, p38, and JNK (Fig. 3A), indicating that RVD5 broadly attenuates α-MSH-induced MAPK activation rather than selectively affecting a single MAPK branch. To determine which MAPK pathway contributes most directly to extracellular melanin production under these experimental conditions, specific inhibitors were employed. Among the ERK1/2 inhibitor PD98059, p38 inhibitor SB203580, and JNK inhibitor SP600125, only SB203580 significantly suppressed extracellular melanin levels in α-MSH-stimulated cells (Fig. 3B). We further analyzed melanogenesis-related proteins following SB203580 treatment. p38 inhibition was associated with reduced protein levels of β-catenin, MITF, and tyrosinase (Fig. 3C). These results suggest that, although RVD5 modulates multiple MAPK pathways, p38 may represent a functionally dominant MAPK branch involved in α-MSH-induced melanin production in this model.

### RVD5 Reduces Intracellular Oxidative Stress and Attenuates ROS-Associated Melanogenic Signaling in α-MSH-Stimulated B16F10 Cells

**Fig. 4:**
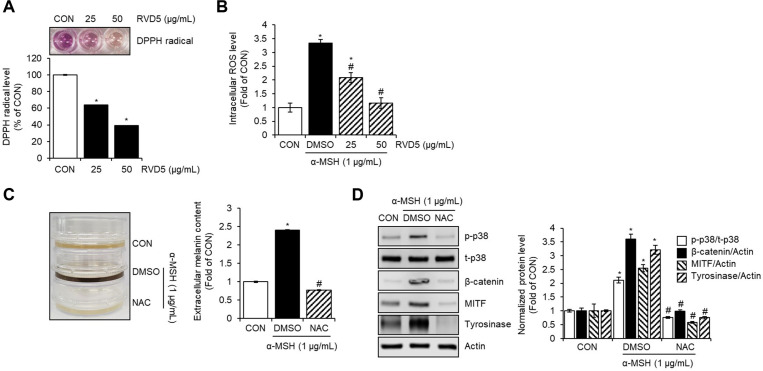
Involvement of ROS in RVD5-mediated anti-melanogenic effects in α-MSH-stimulated B16F10 cells. (**A**) Cell-free antioxidant activity of RVD5 was evaluated using the DPPH radical scavenging assay. (**B**) B16F10 cells were pretreated with RVD5 for 2 h and then co-treated with α-MSH (1 μg/mL) for 48 h. Intracellular ROS was evaluated using the Intracellular ROS Assay Kit according to the manufacturer's protocol. (**C**) B16F10 cells were pretreated with NAC (ROS inhibitor, 10 mM) for 2 h and then co-treated with α-MSH (1 μg/mL) for 48 h. Extracellular melanin content was analyzed by measuring the absorbance of the cell culture medium at 405 nm. (**D**) B16F10 cells were pretreated with NAC (ROS inhibitor, 10 mM) for 2 h and then co-treated with α-MSH (1 μg/mL) for 48 h. The protein levels of p-p38, p38, β-catenin, MITF, and tyrosinase were analyzed using Western blot analysis. * *P* < 0.05 vs. CON (untreated group). ^#^ *P* < 0.05 vs. DMSO (α-MSH-only treated group).

To investigate whether RVD5 exerts antioxidant effects that contribute to its anti-melanogenic activity, we first assessed its radical-scavenging capacity using the DPPH assay. RVD5 dose-dependently scavenged DPPH radicals (Fig. 4A). We next examined intracellular oxidative stress in α-MSH–stimulated B16F10 cells. RVD5 significantly reduced intracellular ROS levels in a concentration-dependent manner (Fig. 4B). To determine whether ROS plays a functional role in melanin production, we treated cells with the ROS scavenger NAC. NAC treatment significantly suppressed extracellular melanin production in α-MSH-stimulated B16F10 cells (Fig. 4C). We further evaluated the impact of ROS inhibition on melanogenesis-related signaling. NAC reduced the phosphorylation of p38 MAPK and decreased the protein levels of β-catenin, MITF, and tyrosinase (Fig. 4D).

## Discussion

Excessive melanin production is a hallmark of hyperpigmentation disorders such as melasma and post-inflammatory hyperpigmentation [15], which present therapeutic challenges due to their chronicity and recurrence [16]. While several depigmenting agents are in clinical use, many are associated with safety concerns or insufficient mechanistic targeting, necessitating the discovery of alternative compounds with high efficacy and low toxicity profiles [4, 5].

In this study, we demonstrated that RVD5 significantly suppresses both extracellular and intracellular melanin synthesis in α-MSH-stimulated B16F10 cells without compromising cell viability. This finding suggests that RVD5 exerts a specific anti-melanogenic effect rather than a general cytotoxic response. Recent studies highlight the tissue-protective and anti-oxidative roles of RVD5 in skin, such as UVB-exposed mouse models, where RVD5 reduced oxidative stress, inflammation, and tissue damage [14]. We believe our findings extend these protective effects into the realm of melanin regulation, positioning RVD5 uniquely as both a skin-protective and depigmenting agent. These properties enhance the value of RVD5 as a potential cosmeceutical or dermatological agent requiring long-term topical application for chronic pigmentary disorders. In summary, our findings indicate that RVD5 is a non-cytotoxic anti-melanogenic candidate in B16F10 cells that effectively suppresses α-MSH-induced melanogenesis while preserving cell viability.

In this study, we demonstrated that RVD5 effectively suppresses melanogenesis in α-MSH-stimulated B16F10 cells by modulating key components of the transcriptional regulatory axis governing melanin biosynthesis. Notably, RVD5 reduced both mRNA and protein levels of tyrosinase, resulting in a significant inhibition of its enzymatic activity. As tyrosinase functions as the rate-limiting enzyme in melanogenesis [17], its precise regulation at both the transcriptional and translational levels is critical for controlling pigment production [18]. Thus, the inhibitory effects of RVD5 are not attributable to cytotoxicity but rather reflect selective targeting of melanocyte-specific pathways. Notably, the suppression of tyrosinase was accompanied by a concomitant reduction in the protein level of the upstream transcription factor MITF. As the master regulator of melanogenesis-related genes, MITF orchestrates pigmentary responses to external stimuli such as α-MSH and ultraviolet radiation [19]. The downregulation of MITF by RVD5 therefore suggests that this compound exerts its inhibitory effect not only on enzymatic activity but also at the transcriptional regulatory level of the melanogenic pathway. Further examination of β-catenin revealed that RVD5 reduced its protein expression under α-MSH-stimulated conditions. Because Wnt/β-catenin signaling is known to promote MITF transcription and thereby stimulate melanogenesis [20, 21], reduced β-catenin may contribute to the downregulation of MITF and tyrosinase. However, the present study did not directly determine whether RVD5 regulates canonical Wnt signaling, β-catenin transcription, or GSK3β-dependent β-catenin degradation. In addition, the decrease in β-catenin protein does not necessarily indicate accelerated protein degradation, because β-catenin may also be regulated at the transcriptional, post-transcriptional, or translational level. Therefore, these findings should be interpreted as evidence that RVD5 suppresses β-catenin-associated melanogenic signaling rather than direct proof that RVD5 acts at an early stage of the Wnt/β-catenin pathway. Future studies examining β-catenin mRNA expression, GSK3β phosphorylation, β-catenin phosphorylation/ubiquitination, and the effects of GSK3β inhibitors such as BIO, LiCl, or SB-216763 will be required to clarify whether RVD5 promotes β-catenin destabilization through Wnt/GSK3β-related mechanisms. Collectively, these results suggest that RVD5 inhibits melanogenesis by modulating the β-catenin/MITF/tyrosinase regulatory pathway without reducing cell viability.

In addition, our results highlight the involvement of MAPK signaling in RVD5-mediated anti-melanogenic activity. Members of the MAPK family, including ERK1/2, p38, and JNK, are well established as key mediators that transmit external cues such as α-MSH into transcriptional activation of MITF and subsequent induction of melanogenic enzymes [22]. In the present study, RVD5 dose-dependently inhibited α-MSH-induced phosphorylation of ERK1/2, p38, and JNK. Therefore, the anti-melanogenic effect of RVD5 should not be interpreted as resulting from selective inhibition of p38 MAPK alone. Rather, RVD5 appears to broadly attenuate α-MSH-induced MAPK activation. To assess the functional contribution of individual MAPK branches to melanin production, we used pharmacological inhibitors of ERK1/2, p38, and JNK. Among these inhibitors, only SB203580 significantly reduced α-MSH-induced extracellular melanin production, suggesting that p38 is the functionally dominant MAPK branch contributing to melanin output under our experimental conditions. This interpretation is consistent with previous evidence showing that p38 activation is involved in MITF expression and melanogenic enzyme regulation [23]. In addition, p38 inhibition was associated with decreased protein levels of β-catenin, MITF, and tyrosinase. Although the inhibitory effect of SB203580 on melanogenesis has been reported previously, SB203580 was used in this study as an internal pharmacological reference to determine whether p38 inhibition produces downstream molecular changes similar to those induced by RVD5. Thus, the added value of Fig. 3C lies not in demonstrating a new function of p38 itself, but in supporting the mechanistic interpretation that RVD5-mediated suppression of melanogenesis is associated, at least in part, with p38-related downregulation of β-catenin, MITF, and tyrosinase. These findings support a functional association between p38 activity and the β-catenin/MITF/tyrosinase melanogenic regulatory pathway, although they do not establish p38 as the exclusive target of RVD5. The concomitant reduction of ERK1/2 and JNK phosphorylation by RVD5 may reflect broader redox-sensitive MAPK modulation, possibly secondary to its ROS-scavenging activity. Alternatively, these changes may represent broader or non-specific signaling modulation by RVD5. Further studies using pathway-specific activation or rescue approaches will be required to clarify the relative contributions of ERK1/2, p38, and JNK to the anti-melanogenic action of RVD5.

Furthermore, oxidative stress emerged as an additional regulatory target of RVD5. Oxidative stress is a well-known driver of melanogenesis, promoting pigment production by activating MAPK pathways and transcription factors such as MITF [24, 25]. ROS, whether generated endogenously or induced by external stimuli such as α-MSH, are recognized for their role in amplifying melanogenic signaling [26]. In the present study, RVD5 demonstrated potent antioxidant activity, as evidenced by its DPPH radical-scavenging capacity and its ability to reduce intracellular ROS levels in α-MSH-stimulated B16F10 cells. These findings suggest that the anti-melanogenic effect of RVD5 is closely associated with its suppression of oxidative stress. Supporting this, treatment with NAC, a representative ROS scavenger, significantly inhibited α-MSH-induced extracellular melanin production while concurrently suppressing phosphorylation of p38 MAPK and decreasing protein expression of β-catenin, MITF, and tyrosinase. Although the suppressive effect of ROS scavenging on melanogenesis has been reported previously, NAC was used in this study as an internal pharmacological reference to determine whether ROS inhibition produces downstream molecular changes similar to those induced by RVD5. Thus, the added value of Fig. 4D lies not in demonstrating a new role of ROS itself, but in supporting the mechanistic interpretation that RVD5-mediated suppression of melanogenesis is associated, at least in part, with ROS-related attenuation of p38 phosphorylation and β-catenin/MITF/tyrosinase expression. These results support the involvement of ROS as upstream modulators of p38-associated melanogenic signaling. Accordingly, the inhibitory effect of RVD5 on melanogenesis may be partly attributable to reduced intracellular ROS, which could attenuate redox-sensitive MAPK activation and downstream melanogenic signaling. However, because ROS-restoration experiments using ROS-generating conditions were not performed, further studies will be needed to determine whether enhancement of ROS generation can attenuate the anti-melanogenic effect of RVD5. Nevertheless, because α-MSH initiates melanogenic signaling through MC1R-dependent cAMP accumulation and subsequent PKA/CREB-mediated MITF induction [26], we cannot exclude the possibility that RVD5 may also act at this early receptor-proximal step. Therefore, direct measurements of cAMP production, PKA/CREB activation, and MC1R-related signaling will be required to determine whether RVD5 suppresses melanogenesis upstream of ROS/MAPK regulation.

## Conclusion

In conclusion, this study demonstrated that RVD5 effectively suppresses α-MSH-induced melanogenesis in B16F10 cells without affecting cell viability. Mechanistically, RVD5 reduced intracellular ROS levels, broadly attenuated α-MSH-induced MAPK activation, and downregulated the β-catenin/MITF/tyrosinase melanogenic regulatory pathway. Pharmacological inhibitor analysis further suggested that p38 is a functionally dominant MAPK branch contributing to melanin production under the present experimental conditions. These findings suggest that RVD5 may be further evaluated as a promising candidate for managing hyperpigmentation or as a functional skin-whitening agent. However, this study did not directly examine whether RVD5 affects the early α-MSH/MC1R/cAMP/PKA/CREB signaling cascade, and further studies will be required to determine whether RVD5 acts upstream of ROS/MAPK regulation. In addition, this study is limited to cell-based in vitro experiments. To further validate the safety, efficacy, and practical applicability of RVD5, future studies should extend these findings to more physiologically relevant models, including 3D human skin equivalents and in vivo animal models. Such studies will be important to determine whether the anti-melanogenic effects of RVD5 observed in B16F10 cells can be reproduced in complex skin microenvironments and to further substantiate its dermatological and cosmeceutical potential.
